# Physical inactivity in nine European and Central Asian countries: an analysis of national population-based survey results

**DOI:** 10.1093/eurpub/ckab028

**Published:** 2021-08-18

**Authors:** Stephen Whiting, Romeu Mendes, Karim Abu-Omar, Peter Gelius, Anna Crispo, Karen McColl, Phillipa Simmonds, Natalia Fedkina, Dianne Andreasyan, Hagverdiyev Gahraman, Tatyana Migal, Lela Sturua, Galina Obreja, Zulfinissio Abdurakhmanova, Ibraeva Nurgul Saparkulovna, Toker Erguder, Banu Ekinci, Bekir Keskinkilic, Shukhrat Shukurov, Rustam Yuldashev, Nino Berdzuli, Ivo Rakovac, Joao Breda

**Affiliations:** 1 WHO European Office for the Prevention and Control of Noncommunicable Diseases, Moscow, Russian Federation; 2 EPIUnit—Instituto de Saúde Pública, Universidade do Porto, Porto, Portugal; 3 Department of Sport Science and Sport, FAU, Erlangen, Germany; 4 Epidemiology and Biostatistics Unit, Istituto Nazionale Tumori-IRCCS-Fondazione G. Pascale, Napoli, Italy; 5 National Institute of Health, National Health Information Analytic Centre, National Institute of Health, Yerevan, Armenia; 6 Public Health and Reforms Center of the Ministry of Health, Baku, Azerbaijan; 7 Department of Health Care Organization of the Ministry of Health, Minsk, Belarus; 8 National Center for Disease Control and Public Health, Tbilisi, Georgia; 9 Department of Social Medicine and Health Management, State University of Medicine and Pharmacy, Chisinau, Republic of Moldova; 10 Republican Nutrition Center, Dushanbe, Tajikistan; 11 Organization of Medical Care and Medicines Policy, Bishkek, Kyrgyzstan; 12 WHO Country Office in Turkey, Ankara, Turkey; 13 General Directorate of Public Health, Ministry of Health, Ankara, Turkey; 14 Central Bureau for the implementation of the Health-3 project, Tashkent, Uzbekistan; 15 WHO Regional Office for Europe, Copenhagen, Denmark

## Abstract

**Background:**

Physical inactivity is a major risk factor for non-communicable diseases. However, recent and systematically obtained national-level data to guide policy responses are often lacking, especially in countries in Eastern Europe and Central Asia. This article describes physical inactivity patterns among adults in Armenia, Azerbaijan, Belarus, Georgia, Kyrgyzstan, Republic of Moldova, Tajikistan, Turkey and Uzbekistan.

**Methods:**

Data were collected using the Global Physical Activity Questionnaire drawing nationally representative samples of adults in each country. The national prevalence of physical inactivity was calculated as well as the proportional contribution to total physical activity (PA) during work, transport and leisure-time. An adjusted logistic regression model was applied to analyze the association of age, gender, education, household status and income with physical inactivity.

**Results:**

National prevalence of physical inactivity ranged from 10.1% to 43.6%. The highest proportion of PA was registered during work or in the household in most countries, whereas the lowest was during leisure-time in all countries. Physical inactivity was more likely with older age in eight countries, with female gender in three countries, and with living alone in three countries. There was no clear pattern of association with education and income.

**Conclusion:**

Prevalence of physical inactivity is heterogeneous across the region. PA during leisure-time contributes minimally to total PA in all countries. Policies and programs that increase opportunities for active travel and leisure-time PA, especially for older adults, women and people living alone will be an essential part of strategies to increase overall population PA.

## Introduction

The countries of Eastern Europe and Central Asia face a rising burden of non-communicable diseases (NCDs) not very different from that seen globally.^1^ NCDs associated with risk factors such as tobacco use, consumption of alcohol and harmful dietary patterns are prevalent across the region.^2–6^ Physical inactivity is another major risk factor for NCDs, in particular, cardiovascular diseases, type 2 diabetes, cancer, obesity and dementia.^7^^,^^8^

Surveillance of the nature and quantity of a population’s physical activity (PA) levels is a valuable tool to guide the development and evaluation of public health policies and interventions designed to prevent NCDs related to physical inactivity.^9^ For adults aged 18–64, the World Health Organization (WHO) recommends 150 min of moderate-intensity aerobic PA per week, or 75 min of vigorous-intensity PA, or an equivalent combination of both, which is equal to at least 600 metabolic equivalents (MET) minutes per week.^10^

Several socioeconomic determinants have been associated with physical inactivity such as age,^11^ gender,^12^ education,^13^ household status^14^ and income.^15^ Furthermore, a country’s level of economic development, culture and availability of infrastructure may have varying influences on the PA levels of different segments of the population.^16^

Total PA may comprise a range of different activities from recreational sports and exercise to active travel such as walking or cycling or PA at work or in the household. These activities can be categorized into three domains—leisure-time, transport and work/household PA—and PA in each of these domains can have different effects depending on many contextual factors.^17^ Leisure-time PA generally contributes the least to total PA^18^ and is lower in low-income countries compared with high-income countries.^19^ The contribution of transport PA appears to be higher among women compared with men in some contexts,^18^ and work/household PA is thought to decrease as a country develops economically and the nature of occupations change.^20^ National level data on the contribution of PA in each of these domains can therefore be useful to guide the prioritization of policy actions.

Investing in policies that enable and promote PA can directly contribute to the achievement of the United Nations’ 2030 Sustainable Development Goals^21^ as well as implementation of the WHO’s Global Action Plan for Physical Activity 2018–2030^22^ and the Physical Activity Strategy for the WHO European Region 2016–2025,^23^ which specifically call for countries to strengthen the monitoring and surveillance of PA to guide national policy action. Some countries in the WHO European region, particularly in Eastern European and Central Asian countries, utilize the WHO STEPwise approach to NCD risk factor surveillance (STEPS) survey to collect information on population health status and NCD risk factors among adults.^24^ The countries included in this study previously lacked data on NCD risk factors including PA, and the WHO Regional Office for Europe provided technical support for implementation of the survey as part of the overall implementation of regional and global strategies related to NCDs.

This study aims to describe the prevalence of physical inactivity among adults in nine Eastern European and Central Asian countries (Armenia, Azerbaijan, Belarus, Georgia, Kyrgyzstan, Republic of Moldova, Tajikistan, Turkey and Uzbekistan) using data collected through recent national STEPS surveys and analyze whether age, gender, education, household status and income were determinants of physical inactivity in this context. It also aims to provide new insights that national and local policymakers may use to priorities policies and interventions designed to increase overall population levels of PA.

## Methods

### Study design and sampling

Data were collected through national STEPS surveys of NCD risk factors. All countries that had collected data on PA across three domains through a national STEPS survey in the WHO European Region from 2013 to 2017 were included in the analysis: Armenia, Azerbaijan, Belarus, Georgia, Republic of Moldova, Kyrgyzstan, Tajikistan, Turkey and Uzbekistan.

The study population was adults aged 15–69 years in Turkey, 18–64 years in Uzbekistan, 25–64 years in Kyrgyzstan and 18–69 years in all other countries. The different study populations were due to the way data were collected in each country. In Turkey, respondents aged 15–17 were assessed according to the WHO recommendations for adults and included in the 18–29-year age group.

The STEPS survey methods are described in depth in the WHO STEPS manual.^24^ Briefly, it uses a multi-stage cluster design with probability proportional to size sampling and has three components: an interviewer-administered questionnaire (STEP 1); anthropometric measurements (STEP 2) and biochemical analyses (STEP 3). Each step has core and expanded questions plus optional modules on topics of interest like oral health or cervical cancer, which can be added depending on country priorities and availability of resources. Data collectors were trained by WHO experts before being sent to the field for data collection.

The required minimum sample size for each country was calculated using the following formula and parameters

$$n=\frac{Z^{2}\times P\times \left(1-P\right)}{d^{2}}$$

where *Z* = level of confidence measure (1.96); *P* = baseline level of indicators (used a value of 0.5, which is closed to expected prevalence of overweight of 50% and results in the largest sample size) and *d* = margin of error (0.05).

A nationally representative sample was drawn for each country. Primary, secondary and tertiary sampling units represented geographical divisions (e.g. provinces, regions and villages). A limited number of households (usually 12–20) in each primary selection unit were selected, and one eligible individual per household was randomly selected to participate in the survey. Participation was voluntary, and informed consent was obtained for STEPS 1 and 2 (combined) and for STEP 3 (separately) of the survey using country-specific language forms. In addition, to protect the confidentiality of collected and archived data, records were anonymized with unique identifiers assigned to participants. Ethical approval was obtained in each country before survey implementation.

### Data collection instrument

The STEPS survey instrument was used for data collection.

In STEP 1, the information was collected through face-to-face interviews and recorded on tablets connected with an online server. During the interview, participants were asked about their sociodemographic characteristics including age, gender, level of education, household status and monthly income and also about behavioural risk factors, including diet, PA, tobacco use and alcohol consumption. Body mass index (BMI) was measured as part of STEP 2, immediately after the interview was completed. The survey instrument was translated from English into national languages and back-translated into English or Russian.

The STEPS instrument includes the Global Physical Activity Questionnaire (GPAQ) to collect information on PA; it is described in depth elsewhere.^25^ The GPAQ comprises 16 items that enable the quantification of PA levels. The instrument was developed to estimate total weekly time spent on PA reported in minutes per week split in three different domains: at work, for transport purposes and as part of leisure-time activities. The duration and frequency of PA (min/week) across the three domains was recorded through the questionnaire in two intensity levels: moderate (4 METs) and vigorous (8 METs). An estimated total moderate-to-vigorous PA in MET-minutes per week was then calculated.^26^ In this study, the proportion of the population that obtained less than 600 MET-minutes per week was classified as physically inactive according to WHO recommendations.^27^

### Data analysis

The prevalence of physical inactivity was estimated as a percentage, and 95% confidence intervals (CI) were calculated. The results were weighted appropriately for multi-stage cluster design and non-response, also taking into account the age and sex distribution of the population, so that the figures presented are nationally representative, and Chi-squared analysis was conducted to test for statistical significance. Key indicators and statistics, tabulations and graphics were produced with the EpiInfo 3.5 software and specific programs developed by WHO.

Logistic regression models analyzed the association of physical inactivity with age (18–29; 30–44; 45–59; 60–69 years), gender (men; women), education (no formal schooling, less than primary school, or primary school completed = <6 years of education; secondary school completed or high school completed = 6–12 years of education; college/university completed or post graduate degree = >12 years of education), household status (living alone; not living alone) and monthly income (quartiles: Q1 = lowest, Q4 = highest). The significance of the trends in risk was assessed by the Wald chi-squared test. Three models were developed: both genders combined and for women and men separately. All models were adjusted for BMI. These data analyses were conducted using the statistical software IBM SPSS Statistics (version 25). *P*-values were set at 0.05.

## Results

Participants included 36 749 adults taken from nationally representative samples for all countries involved. Between countries, the number of respondents varied from 2623 in Kyrgyzstan to 8650 in Turkey. In each national survey, proportionally, more women participated than men (table 1).

**Table 1:** Characteristics of study population, by country

Country	Survey year	Age group (years)	Sample size
Armenia	2016	18–69	5600
Azerbaijan	2017	18–69	2881
Belarus	2016	18–69	5760
Georgia	2016	18–69	5554
Kyrgyzstan	2013	25–64	2623
Republic of Moldova	2013	18–69	5760
Tajikistan	2016	18–69	2881
Turkey	2017	15–69	8650
Uzbekistan	2013	18–64	4350

Prevalence of physical inactivity varied by country ranging from 10.1% to 43.6%. PA during leisure time was the lowest in all countries, and PA was mostly from work or in the household. Analysis of socioeconomic determinants showed that the likelihood of physical inactivity increased with age and was more likely among women compared with men in eight countries and people living alone compared with people not living alone in three countries.

### Prevalence of physical inactivity

Weighted prevalence of physical inactivity varied considerably across countries (table 2). The total prevalence for both genders ranged from 10.1% (95% CI [8.6–11.5]) in the Republic of Moldova to 43.6% (95% CI [41.8–45.4]) in Turkey. For men, total prevalence was lowest in Kyrgyzstan with 8.9% (95% CI [6.4–11.3]) and highest in Turkey 33.1% (95% CI [30.5–35.6]). Among women, prevalence ranged from 9.4% (95% CI [7.7–11.1]) in the Republic of Moldova to 53.9% (95% CI [51.6–56.3]) in Turkey.

**Table 2:** Weighted-prevalence of physical inactivity and composition of physical activity in different domains for both genders, men and women (%, 95% confidence interval)

	ARM	AZB	BLR	GEO	KGZ	MDA	TJK	TUR	UZB	*P*-value
Physical inactivity										
Both genders	21.3 (18.4–24.1)	19.1 (15.9–22.3)	13.2 (11.5–14.8)	17.4 (15.6–19.2)	11.4 (9.6–13.3)	10.1 (8.6–11.5)	28.3 (24.5–32.1)	43.6 (41.8–45.4)	16.4 (13.6–19.1)	<0.0001^*^
Men	22.0 (18.0–26.1)	19.1 (15.3–22.9)	12.8 (10.7–14.9)	16.2 (13.6–18.9)	8.9 (6.4–11.3)	10.7 (8.5–12.9)	17.6 (13.6–21.5)	33.1 (30.5–35.6)	10.6 (7.9–13.3)	<0.0001^*^
Women	20.4 (17.3–23.5)	19.1 (15.5–22.6)	13.5 (11.5–15.5)	18.4 (16.3–20.4)	14.1 (11.7–16.5)	9.4 (7.7–11.1)	39.9 (34.4–45.4)	53.9 (51.6–56.3)	22.5 (18.9–26.2)	<0.0001^*^
Work-related PA										
Both genders	47.4 (44.5–50.3)	55.3 (53.5–57.1)	56.9 (54.8–59.0)	63.4 (61.4–65.4)	69.4 (67.2–71.6)	68.6 (66.8–70.3)	44.2 (40.7–47.7)	55.6 (52.8–58.4)	63.4 (61.5–65.2)	<0.0001^*^
Men	62.9 (57.9–67.9)	53.9 (51.0–56.8)	62.3 (59.4–65.2)	61.9 (58.2–65.6)	70.0 (66.4–73.6)	70.9 (68.2–73.6)	49.6 (44.5–54.7)	62.0 (58.4–65.6)	62.1 (59.3–64.9)	<0.0001^*^
Women	46.6 (43.2–50.0)	56.5 (54.2–58.8)	51.7 (48.7–54.7)	64.2 (61.8–66.6)	68.9 (66.0–71.8)	66.9 (64.5–69.3)	38.5 (33.7–43.3)	44.3 (40.0–48.6)	65.2 (62.8–67.6)	<0.0001^*^
Transport-related PA										
Both genders	49.3 (46.8–51.8)	38.2 (36.8–39.5)	37.3 (35.9–38.8)	34.3 (32.7–35.9)	28.3 (26.4–30.2)	29.5 (28.1–30.9)	45.8 (41.9–49.7)	35.9 (34.5–37.3)	26.0 (24.4–27.6)	<0.0001^*^
Men	33.6 (29.3–37.9)	39.8 (37.6–42.0)	32.2 (30.2–34.2)	33.7 (30.8–36.6)	26.8 (23.7–30.0)	26.5 (24.3–28.7)	38.3 (35.0–41.6)	29.8 (27.7–31.9)	23.9 (21.5–26.3)	<0.0001^*^
Women	50.7 (47.7–53.7)	36.9 (35.1–38.7)	42.1 (40.2–44.0)	34.5 (32.6–36.4)	29.6 (27.2–32.0)	31.7 (29.9–33.5)	53.7 (50.6–56.8)	47.0 (45.0–49.0)	28.5 (26.3–30.7)	<0.0001^*^
Leisure-time PA										
Both genders	3.3 (1.2–5.4)	6.5 (5.1–7.9)	5.8 (4.5–7.1)	2.3 (0.6–4.0)	2.3 (0.8–3.8)	1.9 (0.7–3.0)	10.0 (6.0–14.0)	8.4 (6.4–10.3)	10.6 (8.9–12.2)	<0.0001^*^
Men	3.5 (0.0–7.0)	6.3 (4.3–8.3)	5.4 (3.5–7.3)	4.4 (1.3–7.5)	3.2 (0.7–5.7)	2.6 (0.7–4.5)	12.1 (8.4–15.8)	8.3 (5.8–10.8)	13.9 (11.5–16.3)	<0.0001^*^
Women	2.7 (0.4–5.0)	6.7 (4.7–8.7)	6.2 (4.5–7.9)	1.3 (0.0–2.6)	1.6 (0.0–3.4)	1.4 (0.01–2.8)	7.8 (4.2–11.4)	8.7 (5.8–11.6)	6.3 (4.2–8.4)	<0.0001^*^

*P*-values for the statistical significance of the differences between countries assessed by the chi-squared test.

ARM: Armenia; AZB: Azerbaijan; BLR: Belarus; GEO: Georgia; KGZ: Kyrgyzstan; MDA: Moldova; TJK: Tajikistan; TUR: Turkey; UZB: Uzbekistan; PA: physical activity.

* Statistically significant value for *P *<* *0.05.

### Composition of total PA by domain (work, transport and leisure-time)

Table 2 also shows the composition of total PA from the three domains—work, transport and leisure-time. In most (seven out of nine) countries, the highest proportion of PA (both genders combined) was at work, contributing between 44.2% of total PA in Tajikistan to 69.4% in Kyrgyzstan, whereas transport-related PA was the primary contributor to total PA in Armenia (49.3%) and Tajikistan (45.8%). For both genders and across all populations, time spent on PA during leisure-time was low ranging from 1.9% in the Republic of Moldova to 10.0% in Uzbekistan. For men, work-time PA was the biggest contributor in all countries, and little of their PA was during leisure-time. This was also the case for women in six of the nine countries, whereas in the other three (Armenia, Tajikistan, Turkey), transport-related PA was the biggest contributor.

### Socioeconomic determinants of physical inactivity

Table 3 presents the results of the adjusted logistic regression analysis for both genders combined. In eight out of nine countries (except for Armenia), a significant upward trend on the risk of being physically inactive with increasing age was observed. Regarding gender, in three countries (Tajikistan, Turkey, and Uzbekistan), women were more likely to be physically inactive than men. The association between the number of years of education and physical inactivity varied depending on the country. In Armenia, Republic of Moldova, Tajikistan, and Uzbekistan, the lowest education level was associated with physical inactivity. In Belarus, those with more years of education were more likely to be physically inactive. In three of the nine countries (Armenia, Azerbaijan and Georgia), people that were living alone were more likely to be physically inactive than people not living alone. For monthly income, results were also dependent on the country. For Azerbaijan and Turkey, a significant increasing trend on the risk of being physically inactive with increasing income was observed. For Armenia, the trend was opposite, as the risk of being physically inactive decreased with higher incomes.

**Table 3:** Adjusted logistic regression model for physical inactivity and socioeconomic determinants

		ARM	AZB	BLR	GEO	KGZ	MDA	TJK	TUR	UZB
OR (95% CI)	Age									
	18–29^a^	1	1	1	1	1	1	1	1	1
	30–44	1.02 (0.70–1.49)	0.94 (0.74–1.20)	1.07 (0.79–1.45)	0.71 (0.48–1.14)	1.22 (0.73–2.04)	0.98 (0.70–1.39)	1.01 (0.69–1.46)	0.99 (0.77–1.22)	0.77 (0.61–0.97)
	45–59	1.14 (0.78–1.66)	1.02 (0.80–1.29)	1.11 (0.89–1.50)	1.13 (0.76–1.69)	1.60 (0.96–2.66)	1.08 (0.78–1.50)	0.95 (0.65–1.39)	1.04 (0.83–1.30)	1.22 (0.95–1.57)
	60–69	1.40 (0.94–2.08)	1.78 (1.37–2.31)	2.47 (1.82–3.34)	1.55 (1.03–2.33)	2.95 (1.66–5.24)	1.79 (1.29–2.50)	2.74 (1.74–4.31)	1.91 (1.48–2.47)	2.42 (1.61–3.63)
	*P*-value	0.2	<0.0001^*^	<0.0001^*^	0.001^*^	<0.0001^*^	<0.0001^*^	<0.0001^*^	<0.0001^*^	<0.0001^*^
OR (95% CI)	Gender									
	Men^a^	1	1	1	1	1	1	1	1	1
	Women	0.81 (0.63–1.05)	1.01 (0.86–1.16)	0.89 (0.76–1.06)	1.07 (0.85–1.36)	1.26 (0.94–1.71)	0.84 (0.69–1.03)	1.76 (1.37–2.27)	2.15 (1.86–2.50)	2.11 (1.75–2.56)
	*P*-value	0.1	0.9	0.2	0.5	0.1	0.09	<0.0001^*^	<0.0001^*^	<0.0001^*^
OR (95% CI)	Education									
	<6 years^a^	1	1	1	1	1	1	1	1	1
	6–12 years	0.47 (0.21–1.01)	0.88 (0.59–1.33)	0.73 (0.59–0.89)	0.92 (0.45–1.88)	0.99 (0.64–1.56)	0.69 (0.53-0.88)	0.42 (0.30–0.60)	0.93 (0.75–1.16)	1.16 (0.87–1.55)
	>12 years	0.36 (0.16–0.80)	0.83 (0.55–1.25)	1.30 (1.03–1.65)	1.00 (0.49–2.04)	1.20 (0.71–2.02)	0.83 (0.60–1.14)	0.38 (0.26–0.55)	0.85 (0.66–1.08)	0.81 (0.60–1.09)
	*P*-value	0.02	0.5	<0.0001^*^	0.7	0.6	0.008^*^	<0.0001^*^	0.4	0.001
OR (95% CI)	Household status									
	Not living alone^a^	1	1	1	1	1	1	1	1	1
	Living alone	1.33 (1.04–1.72)	1.19 (1.01–1.41)	0.98 (0.83–1.14)	1.42 (1.13–1.77)	1.15 (0.74–1.58)	1.09 (0.89–1.36)	0.96 (0.68–1.35)	1.09 (0.92–1.28)	0.88 (0.71–1.10)
	*P*-value	0.03^*^	0.04^*^	0.8	0.002^*^	0.4	0.4	0.8	0.3	0.3
OR (95% CI)	Monthly income									
	Q1^a^	1	1	N/A	1	1	1	1	1	N/A
	Q2	1.59 (1.13–2.24)	1.18 (0.96–1.45)	N/A	0.83 (0.62–1.13)	0.81 (0.56–1.17)	1.01 (0.77–1.33)	1.07 (0.76–1.51)	0.91 (0.74–1.11)	N/A
	Q3	1.22 (0.88–1.71)	1.15 (0.93–1.44)	N/A	1.07 (0.80–1.44)	0.84 (0.56–1.27)	0.92 (0.68–1.24)	0.99 (0.67–1.47)	1.30 (1.06–1.60)	N/A
	Q4	0.86 (0.60–1.25)	1.45 (1.19–1.78)	N/A	1.08 (0.80–1.48)	1.20 (0.81–1.77)	0.98 (0.72–1.34)	1.05 (0.74–1.47)	1.39 (1.12–1.73)	N/A
	*P*-value	0.003^*^	0.004^*^		0.4	0.2	0.9	0.9	<0.0001^*^	

ARM: Armenia; AZB: Azerbaijan; BLR: Belarus; GEO: Georgia; KGZ: Kyrgyzstan; MDA: Moldova; TJK: Tajikistan; TUR: Turkey; UZB: Uzbekistan; N/A: not available.

a Reference category; *P*-values for the statistical significance of the trend in risk assessed by the Wald chi-squared test.

* Statistically significant value for *P *<* *0.05; all analyses were adjusted for body mass index.

Table 4 shows the association between the weighted prevalence of physical inactivity and PA and age groups for each country. Physical inactivity increases with age in all countries. In general, PA decreases with increasing age; the highest prevalence of PA is observed among younger age-group,^18–29^ particularly in Tajikistan, Uzbekistan and Turkey. Overall, PA is more prevalent up to 44 years of age while >44 years of age physical inactivity (PIA) is more prevalent.

**Table 4:** Association between meeting World Health Organization physical activity recommendations and age-groups for each country

	ARM		AZB		BLR		GEO		KGZa		MDA		TJK		TURa		UZBa	
Age	PIA	PA	PIA	PA	PIA	PA	PIA	PA	PIA	PA	PIA	PA	PIA	PA	PIA	PA	PIA	PA
18–29	1103 (29.9%)	397 (35%)	194 (32%)	766 (29.8%)	75 (16.7%)	612 (23.7%)	92 (20.3%)	506 (25.2%)	36 (17.3%)	353 (24.7%)	104 (30.5%)	744 (34.6%)	51 (46.5%)	262 (59.4%)	482 (31.2%)	688 (36%)	218 (43.9%)	917 (46.8%)
30–44	136 (26.1%)	494 (30.2%)	262 (29.9%)	1308 (33.3%)	167 (27.1%)	1237 (31.5%)	167 (28.6%)	868 (30.3%)	98 (33.9%)	863 (39.3%)	139 (27.4%)	1094 (29.1%)	84 (27.9%)	261 (23.3%)	727 (32.5%)	975 (32.6%)	269 (21.9%)	1281 (28.7%)
45–59	168 (31.8%)	554 (25.6%)	370 (25.2%)	1780 (28.7%)	232 (28.9%)	1664 (31.2%)	263 (32.7%)	1230 (29.6%)	135 (36.9%)	876 (30%)	231 (29.4%)	1457 (27.3%)	79 (16.5%)	252 (13.6%)	709 (23.1%)	907 (23.5%)	242 (27.4%)	748 (21.7%)
60–69	132 (12.2%)	305 (9.2%)	258 (13%)	664 (8.3%)	234 (27.3%)	770 (13.6%)	238 (18.4%)	786 (14.9%)	59 (11.9%)	200 (6%)	197 (12.7%)	841 (9%)	43 (9.1%)	59 (3.7%)	463 (13.2%)	381 (7.9%)	60 (6.8%)	99 (2.8%)
*P*-value	<0.0001^*^		<0.0001^*^		<0.0001^*^		<0.0001^*^		<0.0001^*^		<0.0001^*^		<0.0001^*^		<0.0001^*^		<0.0001^*^	

*P*-values for the statistical significance of the association between the weighted prevalence of PIA or PA and age groups assessed by the chi-squared test.

ARM: Armenia; AZB: Azerbaijan; BLR: Belarus; GEO: Georgia; KGZ: Kyrgyzstan; MDA: Moldova; TJK: Tajikistan; TUR: Turkey; UZB: Uzbekistan; PA: physical activity; PIA: physical inactivity.

a Age ranges differed: in Turkey the youngest age group included respondents aged 15–17 years and oldest age group included respondents from 66 to 69 years; in Kyrgyzstan, the youngest age group was from 25 to 29 years and oldest age group was from 60 to 64 years; in Uzbekistan the oldest age group was from 60 to 64 years.

* Statistically significant value for *P *<* *0.05.

## Discussion

This study has been the first to provide a systematic and comprehensive analysis of population physical inactivity in Eastern Europe and Central Asia. National prevalence of physical inactivity ranged from 10.1% to 43.6%. In most countries, the proportion of total PA came primarily from work/household PA, followed by transport PA, whereas leisure-time PA contributed the least in all countries, findings which align with data from other regions.^18^ The likelihood of being physically inactive was highest among the oldest age group age in eight countries, female gender in three countries, and people living alone in three countries. For all countries included, these are the first nationally representative prevalence estimates of physical inactivity conducted alongside an assessment of the composition of PA across different domains and analyses of factors associated with physical inactivity using population-based survey data.

### Prevalence of physical inactivity

The results reveal considerable differences in the prevalence of physical inactivity between countries in the east of the WHO European Region. In the Republic of Moldova (10.1%) and Kyrgyzstan (11.4%), only around one in 10 people do not meet the WHO recommendations for PA, and levels of physical inactivity are well below the global average (27.5%) in most countries.^28^ However, almost a third (28.3%) of the population in Tajikistan and almost half (43.6%) of the people in Turkey are physically inactive. These findings are not surprising, as differences in the estimated prevalence of physical inactivity between countries have been demonstrated previously.^28^

These differences in PA between countries may be influenced by the political, social, cultural or natural environments in each country as well as setting-specific characteristics.^29^ The nine countries are at different stages of economic development, with four upper-middle-income countries (Armenia, Azerbaijan, Belarus, Turkey), four lower-middle-income countries (Georgia, Kyrgyzstan, Republic of Moldova, Uzbekistan) and one low-income country (Tajikistan). Globally, physical inactivity tends to decrease as national incomes increase.^20^^,^^30^ On the other hand, comparisons with higher-income countries in the WHO European Region indicate that levels of physical inactivity in the countries included in our study already tend to be rather low. For example, some estimates of physical inactivity using results from the 2017 Eurobarometer survey conducted in the 28 Member States of the European Union, found that the prevalence of physical inactivity ranged from around 23% to 71%, and in seven countries, the prevalence of physical inactivity was over 50%.^31^ Although direct comparisons are always difficult and can only be made with caution, this could be concerning for countries in Eastern Europe and Central Asia as the NCD burden is already comparatively high.^1^ It is therefore possible that an increase in levels of physical inactivity could further exacerbate the existing burden of NCDs in Eastern Europe and Central Asia.

### Composition of physical activity by domain

For the first time, our study described not only the prevalence of physical inactivity in this region but also the composition of PA across three domains, providing insights into the potential effectiveness of current and future strategies to increase PA in each country. In all countries, at least half, and sometimes as much as two-thirds of PA, takes place during work or household work. Active transport—walking and/or cycling—accounts for between a quarter and a half of total PA, and the contribution of leisure-time PA is relatively small (as low as 2–3% in some countries). Consequently, specific strategies that provide continued opportunities and infrastructure to support safe active transport, participation in sports and regular leisure-time PA are needed throughout the region.

It has been suggested that levels of physical inactivity may be related to occupational changes from labor-intensive to sedentary office-based and service-oriented professions.^20^^,^^30^ PA at the workplace ranged from 44.2% to 69.4% of total PA time, and there was a lower proportion of PA during work time in the four upper-middle income countries included (Armenia, Azerbaijan, Belarus, Turkey). As most adults spend most of their time at work, PA-promotional programs aimed at those in sedentary occupations could potentially impact a large proportion of the working population, especially in upper-middle income countries. For the lower income countries included, these findings may represent a glimpse into the future as more occupations become sedentary or mechanized. These findings point to the need for multi-sectoral policies and programs to promote PA across the region, but specific interventions may need to be prioritized depending on the country context.

### Socioeconomic determinants of physical inactivity

Physical inactivity was more likely among older adults in eight out of nine countries, which reflects established global patterns.^28^ Promoting PA among older people is a priority to be addressed due to the lower levels demonstrated here as well as the critical role of PA in maintaining functional capacity and independence, reducing the risk of falls and resulting injuries, and improving general mental health and well-being.^32^ Regular PA for older adults is important for healthy ageing, and the WHO recommends the same minimum level of PA for all adults aged 18–65.^27^ These results point to the need for implementation of policy priorities recommended by WHO/Europe to promote PA among older people, including the provision of quality advice by health professionals, appropriate infrastructure and environments for PA and community-based action to involve older people in social PA.^33^

For Tajikistan, Turkey and Uzbekistan, our data correspond with global evidence that women are more likely to be physically inactive compared with men.^28^ Our results show that in these three countries, women were twice as likely not to meet the WHO recommended levels for PA compared with men, which may be due to specific cultural and gender norms that limit opportunities for women to participate in PA and sports.^34^ Culturally appropriate opportunities for adult women to participate in recreational PA could be developed in partnership with local sports and community organizations. As there has thus far been limited relevant research conducted in this region, these results present an important basis for further investigation into the specific barriers limiting the participation of women in PA and sports to help guide the development of national and local solutions.

The analysis of the likelihood of being physically inactive due to household status, education and income presented a complex picture. In Armenia, Azerbaijan and Georgia, people living alone were more likely to be physically inactive, which may indicate the importance of family and social support for PA, especially for initiating leisure-time PA.^35^ In three countries, physical inactivity appears to decrease at higher income levels, whereas in one country, it appeared that the opposite was true. This could be due to the complex interaction between socioeconomic determinants and PA that are often dynamic as life events, such as marital transitions, entering university or reducing income, may have different impacts on PA levels for men and women of different ages.^36^ The specific interactions between living alone, education or monthly income on total PA may also vary between countries due to cultural differences or the level of economic development, as well as socio-political conditions, built environments or climate.^37^^,^^38^

The promotion of PA as part of daily life to all adults is at the core of the WHO’s Physical Activity Strategy for the WHO European Region 2016–2025, which calls on national and local authorities to promote human-powered transport by establishing a mix of accessible walking and cycling infrastructures and improving the availability and affordability of public transport. Sport-for-all approaches may also be considered to address the generally low levels of PA for leisure or recreation across the region. National governments and local decision-makers could also take concerted action to increase the availability of resources and spaces for recreational PA as well as to maximize participation by addressing issues of affordability, inclusiveness and cultural acceptability with the goal of ensuring more people in countries across the region can meet the WHO recommended levels of PA to protect their health.

### Strengths and limitations

One of the strengths of this study is the use of data collected through the WHO STEPS survey instrument, which uses a standardized protocol that produces nationally representative data. WHO conceptualized the survey and provided close technical assistance for survey planning, implementation and analysis in all nine countries, yielding a large dataset that enables comparisons of NCD risk factors between countries. The PA component of the survey utilizes the GPAQ, which was developed and recommended by WHO for the assessment of population level PA and has been validated previously.^39^ However, its validity is relatively low, and it is challenging to use the same questionnaire across different countries with different data collection teams and control for factors such as the interpretation of the questions, understanding of the intensity of PA and the recall period.^40^ The cross-sectional study design is acknowledged as well as the fact that the data collection took place at different times, in some cases four years apart, so there may have been changes in PA policy, seasonal effects or impact interventions that took effect in certain countries. Another limitation is that the countries included were only those that had conducted this particular survey and are not representative of a single coherent geographic region, so there was no theory-driven logic for the inclusion of these countries and the exclusion of others. In addition, there were some variation in the age ranges used in the youngest age groups in Turkey and Kyrgyzstan and the oldest age group in Uzbekistan, therefore these findings must be viewed with caution.

### Future research

This study presents only an overview and analyses of the data available on physical inactivity from nine STEPS surveys that have been conducted in Eastern Europe and Central Asia. Although it was outside the scope of this study, more granular analyses of national cross-sectional surveys can be conducted to gain additional insights to help guide design and implementation of national and local interventions to increase PA among specific segments of the population. In addition, due to the recognized limitations of self-reporting of PA, it would be useful to further explore the integration of objective PA assessment (e.g. using pedometer or accelerometer devices) into PA surveillance and studies such as the STEPS survey.

## Conclusions

The national prevalence of physical inactivity varies across Eastern Europe and Central Asia. Most PA is from people’s occupations with a low proportion of total PA for leisure or recreation. Older adults, women and people living alone were less likely to achieve recommended levels of PA, but these findings were not uniform across all countries included. Policy action is needed to provide more opportunities for PA during leisure time and enable active transport. Implementation research may help countries to identify effective policies and interventions that can reach the most inactive segments of the population in consideration of the level of development of the country, cultural factors and resources available for PA promotion.
